# Nicotine strength of e‐liquids used by adult vapers in Great Britain: A population survey 2016 to 2024

**DOI:** 10.1111/add.16576

**Published:** 2024-06-19

**Authors:** Sarah E. Jackson, Jamie Brown, Lion Shahab, Deborah Arnott, Linda Bauld, Sharon Cox

**Affiliations:** ^1^ Department of Behavioural Science and Health University College London London United Kingdom; ^2^ SPECTRUM Consortium United Kingdom; ^3^ Behavioural Research UK United Kingdom; ^4^ Action on Smoking and Health United Kingdom; ^5^ Usher Institute University of Edinburgh Edinburgh United Kingdom

**Keywords:** disposable vapes, e‐cigarettes, e‐cigarette tax, vaping, vaping duty, vaping tax

## Abstract

**Background and aims:**

In March 2024, the UK government announced plans to introduce a Vaping Products Duty that will tax e‐liquids based on their nicotine strength. This study examined trends in the nicotine strength of e‐liquids used by adult vapers and differences in those currently used across relevant subgroups.

**Design:**

Nationally‐representative, cross‐sectional household survey, July 2016 to January 2024.

**Setting:**

Great Britain.

**Participants:**

7981 adult vapers.

**Measurements:**

Participants were asked whether the e‐cigarette they mainly use contains nicotine (yes/no) and the e‐liquid strength (no nicotine, >0–≤ 6, 7–11, 12–19 or ≥20 mg/ml). We also collected information on the main device type used (disposable/refillable/pod), age, gender, occupational social grade, history of ≥1 mental health conditions, smoking status and (among past‐year smokers) level of cigarette addiction.

**Findings:**

The proportion of vapers in England using high‐strength (≥20 mg/ml) e‐liquids increased from an average of 3.8% (95% confidence interval [CI] = 2.9–5.0) up to June 2021 to 32.5% (CI = 27.9–37.4) in January 2024 (the vast majority of whom [93.3% in January 2024] reported using exactly 20 mg/ml; the legal limit). This rise was most pronounced among those using disposable e‐cigarettes, those aged 18‐24 years and all smoking statuses (including never smokers) except long‐term (≥1y) ex‐smokers. Of those surveyed in 2022–2024 in Great Britain, overall, 89.5% (CI = 88.1–90.8) said they usually used e‐cigarettes containing nicotine, 8.7% (CI = 7.5–10.0) used nicotine‐free e‐cigarettes, and 1.8% (CI = 1.2–2.4) were unsure. The proportion using ≥20 mg/ml was higher among those mainly using disposable (47.9%) compared with pod (16.3%) or refillable (11.5%) devices; never smokers (36.0%), current smokers (28.8%) or recent (<1y) ex‐smokers (27.4%), compared with long‐term ex‐smokers (13.9%); and younger (16–24y; 44.2%) compared with older (≥25y; range 9.4–25.1%) age groups. There were no notable differences across other subgroups of interest.

**Conclusions:**

Use of high‐strength nicotine e‐liquids in England appears to have increased sharply in recent years. Most adult vapers in Great Britain appear to use e‐cigarettes that contain nicotine but different subgroups use different strengths: nicotine strengths tend to be higher among those who mainly use disposable devices and those aged 16–24y, and lower among long‐term ex‐smokers.

## INTRODUCTION

In Great Britain, the prevalence of e‐cigarette use (vaping) has risen rapidly among adolescents and young adults since 2021 [1, 2, 3]. This has largely been attributed to the introduction of new disposable e‐cigarettes (vapes) to the market [2]. These products are easy to use, have colourful design and branding, come in a variety of flavours and typically contain high levels of nicotine, delivered in palatable nicotine‐salts e‐liquid [4, 5, 6]. They are also cheaper to buy than both cigarettes and refillable e‐cigarettes—one of the most popular brands with underage vapers, Elf Bar 600, can be found online for £2.99 (US$3.80, €3.50) and in convenience stores and supermarkets for £5.99 (US$7.70, €7.00)—making them more affordable for experimental use.

The United Kingdom (UK) government has made reducing youth vaping a key public health policy priority [7]. In January 2024, the Prime Minister announced a ban on disposable e‐cigarettes as part of a package of measures designed to tackle the rise in youth vaping [8, 9]. In March 2024, the Chancellor of the Exchequer announced in the Spring budget that a new Vaping Products Duty would also be introduced from October 2026 in an effort to make vaping less affordable to children [10]. According to the consultation document, which sets out the proposal for how the duty will be designed and implemented, the Vaping Products Duty will be an excise duty levied on the e‐liquid in e‐cigarettes with higher levels of duty applied to higher‐strength nicotine e‐liquids (proposed to be £1 per 10 mL for nicotine‐free e‐liquids, £2 per 10 mL for e‐liquids that contain up to 10.9 mg of nicotine per mL and £3 per 10 mL for e‐liquids that contain 11 mg or more nicotine per mL) [10]. The rationale for the differential tax rate is that ‘given the known harms caused by nicotine addiction, the government's intention is also to encourage consumers to reduce their nicotine intake by switching to lower or nicotine‐free options, further supporting health objectives’ [10]. Some evidence suggests that use of products with higher nicotine strength is associated with greater symptoms of dependence (e.g. frequency of vaping, urges to vape and perceived vaping addiction) among young vapers [11].

Ministers have acknowledged the need to try and strike the right balance with a price increase that acts as a deterrent but ensures vaping remains a more affordable option than smoking to encourage adult smokers to switch to the less harmful product [9]. To that end, the UK government concurrently announced a one‐off increase on the duty on tobacco (by £2 per 100 cigarettes or 50 g of tobacco) in October 2026 to coincide with the introduction of the Vaping Products Duty [10]. However, even if they remain less expensive than tobacco, taxing higher‐strength nicotine e‐liquids at higher rates could have unintended consequences for people who smoke and those who have switched from smoking to vaping. Higher‐strength e‐liquids provide better relief from withdrawal and satisfy cravings for tobacco [12] and may, therefore, be more effective for helping prevent relapse [13]. Making these products more expensive could disincentivise their use and drive vapers toward cheaper, lower‐strength e‐liquids. This could potentially undermine smoking cessation [14] or result in increased use of e‐liquid to compensate [15] (including among young vapers not trying to quit smoking); therefore, increasing potential toxicant exposure and associated risks to health [16]. It could also prompt them to source illicit higher strength products and potentially also to mix their own e‐liquids, which poses potential safety risks [17]. These responses may be more likely among vapers from disadvantaged groups (e.g. those working in lower paid jobs or with mental health conditions), who tend to be more dependent on nicotine [18, 19] and have lower disposable incomes.

Understanding what nicotine strengths adult vapers in Great Britain are currently using, before the introduction of the Vaping Products Duty, and how this differs across subgroups of vapers, can offer insight into who will be most affected by the duty. In line with the European Union Tobacco Products Directive (TPD), the maximum nicotine concentration permitted in e‐liquids for sale as consumer products in Great Britain is 20 mg/mL [16]. A representative survey of adults in England in 2021 indicated the most popular strength of e‐liquid was ≤6 mg/mL (used by 39.9% of vapers), with just 5.4% using 20 mg/mL or more [16], but it is likely that this may have changed since high‐strength disposable e‐cigarettes have become popular. This study aimed to:

1. Estimate trends in the nicotine strength of e‐liquids used among adult vapers in England between 2016 and 2023, overall and by the main device type used, age and smoking status.
2. Provide up‐to‐date descriptive information on the use of different e‐cigarette nicotine strengths among adult vapers in Great Britain in 2022–2024, overall and by the main device type used, vaping frequency, age, gender, socioeconomic position, history of mental health conditions, smoking status and (among past‐year smokers) level of cigarette dependence.
3. Understand which groups would be most affected by the proposed Vaping Products Duty structure.

## METHODS

### Design

Data were drawn from the Smoking Toolkit Study [20, 21]. This is a repeat cross‐sectional survey of adults (≥16 years) that captures a broad range of data on smoking and vaping. It began in England in 2006 (*n* = ~1700 per month) and expanded to cover Wales (*n* = ~300) and Scotland (*n* = ~450) from October 2020. Each month, a new sample is recruited using a hybrid of random probability and quota sampling. Comparisons with other national surveys and sales data indicate that key variables such as socio‐demographic characteristics, smoking prevalence and cigarette consumption are nationally representative [20, 22].

Interviews were conducted face‐to‐face up to the start of the coronavirus disease 2019 (Covid‐19) pandemic. Social distancing restrictions meant no data were collected in March 2020 and data collection pivoted to telephone interviews from April 2020 onward. The two data collection modalities show good comparability. When social distancing restrictions were lifted, we ran a parallel telephone and face‐to‐face survey wave and yielded similar estimates for key socio‐demographic, smoking and nicotine product use measures [23]. Data were not collected from 16‐ and 17‐year‐olds between April 2020 and December 2021.

### Sample selection for the present analysis

For the present analyses, we selected data from two samples of participants who reported current vaping at the time of the survey. The trend analyses focused on respondents in the period from July 2016 (the first wave to assess nicotine strength) to January 2024 (the most recent data at the time of analysis). We restricted this sample to those living in England and age ≥18 for consistency across the time series, given that the Wales and Scotland data collection began in 2020 and 16‐ and 17‐year‐olds were not included in every wave. The analyses of current nicotine strengths used in 2022 to 2024 focused on all respondents living in Great Britain and age ≥16 in the period from January 2022 to January 2024 (the most recent 2 years of data available, after new disposable e‐cigarettes became popular) [2].

Nicotine strength was not assessed in certain waves (May, June, August, September, November and December 2022 and February, March, May, August, September, November and December 2023), so we excluded participants surveyed in these waves from our analytic samples.

### Measures

Vaping status was assessed within several questions asking about use of a range of nicotine products. Current smokers were asked ‘Which, if any, of the following are you currently using to help you cut down the amount you smoke?’ and ‘Do you regularly use any of the following in situations when you are not allowed to smoke?’; current smokers and those who have quit in the past year were asked ‘Can I check, are you using any of the following either to help you stop smoking, to help you cut down or for any other reason at all?’; and non‐smokers were asked ‘Can I check, are you using any of the following?’ Those who reported using an e‐cigarette in response to any of these questions were considered current vapers and formed our analytic sample.

Nicotine strength was assessed with two questions. The first asked: ‘Does the electronic cigarette or vaping device you mainly use contain nicotine?’ with response options ‘yes’, ‘no’ and ‘do not know’. Those who responded yes to this question were then asked: ‘What strength is the e‐liquid that you mainly use in your electronic cigarette or vaping device?’ with response options ‘6 mg/mL (0.6%) or less’, ‘7 mg/mL (0.7%) to 11 mg/mL (1.1%)’, ‘12 mg/mL (1.2%) to 19 mg/mL (1.9%)’, ‘20 mg/mL (2.0%) or more’ and ‘do not know’. From the most recent survey (January 2024), the response option ‘20 mg/mL (2.0%) or more’ has been replaced with ‘20 mg/mL (2.0%)’ and ‘more than 20 mg/mL (2.0%)’ to distinguish between those using e‐liquids with nicotine strengths at versus exceeding the legal limit. The vast majority (*n* = 56/60, 93.3%) of participants surveyed in this wave who reported using 20 mg/mL or more said they used 20 mg/mL exactly (i.e. the maximum legal limit).

Device type was assessed with the question: ‘Which of the following do you mainly use …? ’ Response options were: refillable – ‘An e‐cigarette or vaping device with a tank that you refill with liquids (rechargeable)’ or ‘A modular system that you refill with liquids (you use your own combination of separate devices: batteries, atomizers, etc.)’; disposable – ‘A disposable e‐cigarette or vaping device (non‐rechargeable)’; and pod – ‘An e‐cigarette or vaping device that uses replaceable pre‐filled cartridges (rechargeable)’.

The disposable response option was included in the surveys across the entire period (except for May, June and August 2022, when device type was not assessed), because older disposable devices were available before the new nicotine‐salts disposable products were introduced to the market in 2021.

Vaping frequency was assessed by asking vapers: ‘How many times per day on average do you use your nicotine replacement product or products?’ Response options were: ‘1’, ‘2’, ‘3 to 4’, ‘5 to 7’, ‘8 to 11’, ‘12+’, ‘Not every day but at least once a week’, ‘Not every day and less than once a week’, ‘Do not know’. Those who reported use at least once a day were considered to be vaping daily and those who reported use less than once a day were considered to be vaping non‐daily.

Socio‐demographic characteristics included age (16–24/25–34/35–44/45–54/55–65/≥65 years), gender (men/women), occupational social grade (ABC1 includes managerial, professional and upper supervisory occupations/C2DE includes manual routine, semi‐routine, lower supervisory and long‐term unemployed), nation (England/Wales/Scotland) and history of ≥1 diagnosed mental health condition since the age of 16 (yes/no; assessed between April 2020 and June 2023 among all participants in England and ~50% of participants in Wales and Scotland).

Smoking status was assessed by asking participants, which of the following best applied to them: (a) ‘I smoke cigarettes (including hand‐rolled) every day’, (b) ‘I smoke cigarettes (including hand‐rolled), but not every day’, (c) ‘I do not smoke cigarettes at all, but I do smoke tobacco of some kind (e.g. pipe, cigar or shisha)’, (d) ‘I have stopped smoking completely in the last year’, (e) ‘I stopped smoking completely more than a year ago’, or (f) ‘I have never been a smoker (i.e. smoked for a year or more)’. Those who responded (a) to (c) were considered current smokers, those who responded (d) recent (<1 year) ex‐smokers, (e) long‐term (≥1 year) ex‐smokers and (f) never smokers.

Among current and recent ex‐smokers (past‐year smokers), level of cigarette dependence was assessed with self‐reported ratings of strength of urges to smoke over the past 24 hours (‘not at all’ [coded 0], ‘slight’ [1], ‘moderate’ [2], ‘strong’ [3], very strong [4] and extremely strong [5]). This variable was also coded 0 for smokers who responded ‘not at all’ to the (separate, previous) question: ‘How much of the time have you spent with the urge to smoke?’ (participants who responded ‘not at all’ were not asked how strong their urges had been). This validated measure performs well compared with other established measures of dependence [24].

Our rationale for selecting these variables for analysis was that device type and vaping frequency are key vaping characteristics that might be associated with use of different nicotine strengths; the socio‐demographic variables are known to be associated with vaping and smoking; smoking status is strongly associated with vaping; and level of dependence may influence people's nicotine strength preferences.

### Statistical analysis

Analyses were conducted using R v.4.2.1. They were not pre‐registered and should be considered exploratory.

Survey weights were applied to match the sample to the demographic profile of England or Great Britain, as relevant for the specific analysis [20, 21], with specific weights used for analyses of mental health conditions to account for this variable not being assessed among all participants in Wales and Scotland.

We excluded participants who did not respond to the questions on nicotine strength (those who responded that they did not know were included); those with missing data on other variables (see Table S1 for details) were excluded on a per‐analysis basis (see figure legends and tables for information on sample sizes for each analysis).

#### Trend analyses

Using data from vapers age ≥18 years in England surveyed between July 2016 and January 2024, we used unadjusted logistic regression to test associations (using individual‐level data) between survey wave and the nicotine strength of e‐liquids used. Each response option for nicotine strength, including do not know responses, was dummy coded as one versus else (i.e. no nicotine vs all other responses; 6 mg/mL or less vs all other responses; etc.). Survey wave was modelled using restricted cubic splines with five knots, to allow relationships with time to be flexible and non‐linear. We did not explore seasonality or autocorrelation.

To explore moderation of trends by the main device type used, age and smoking status, we repeated the models including the interaction between the moderator of interest and survey wave —therefore, allowing time trends to differ across subgroups. Each interaction was tested in a separate model. We did not focus on *P*‐values for these interactions, but rather used predicted estimates from these models to plot the proportions (with 95% CI) of vapers using each nicotine strength over time within subgroups. On these figures, we included a vertical line indicating the timing of the rise in popularity of disposable e‐cigarettes among young adults (estimated to be June 2021, based on previous findings) [2, 25] to contextualise changes in the nicotine strengths being used.

#### Analyses of current nicotine strengths used

Using data from vapers age ≥16 years in Great Britain surveyed between January 2022 and January 2024, we reported the proportions (with 95% CI) of vapers who reported using each different nicotine strength or who did not know, overall and by the main device type used, vaping frequency, socio‐demographic characteristics, smoking status and (among past‐year smokers) strength of urges to smoke.

We also calculated these separately stratified by the main device type used, to check whether the pattern of results differed between those mainly using refillable, disposable and pod devices (we excluded strength of urges to smoke from these analyses, because of low numbers within subgroups).

Finally, we used univariate multinomial logistic regression to test associations (among users of all device types) between nicotine strength and each participant characteristic in turn, adjusting only for survey wave. For this analysis, we collapsed nicotine strengths to no nicotine, ≤11 mg/mL nicotine and ≥12 mg/mL nicotine (excluding those who responded that they did not know), to approximate the proposed structure of the Vaping Products Duty [10]. This was intended to offer insight into which groups would be most affected by the proposed duty.

## RESULTS

A total of 9286 vapers were surveyed in eligible waves, of whom 8641 provided data on the nicotine strength of the e‐liquid they mainly used. For the trend analyses, we selected those age ≥18 and living in England, providing a sample of 7314 participants (weighted mean [SD] age = 40.8 [15.2] years; 44.8% women; 54.0% social grades C2DE). For analyses of current nicotine strengths used in 2022 to 2024, we selected those age ≥16 years and living in Great Britain surveyed between January 2022 and January 2024, providing a sample of 2373 participants (weighted mean [SD] age = 37.4 [15.3] years; 47.3% women; 54.1% social grades C2DE). In total, we analysed data from 7981 unique participants (1706 were included in both samples, 5608 in the trend analysis sample only, and 667 in the 2022–2024 sample only). Characteristics of the two samples are shown in Table S1. Vaping characteristics and age stratified by smoking status are shown in Table S2. The majority (>70%) of participants, including never smokers, reported vaping daily. Never smokers were more likely to report using disposable devices and tended to be younger compared with ex‐ and current smokers.

### Trends in nicotine strength of e‐liquids used by vapers in England

**FIGURE 1:**
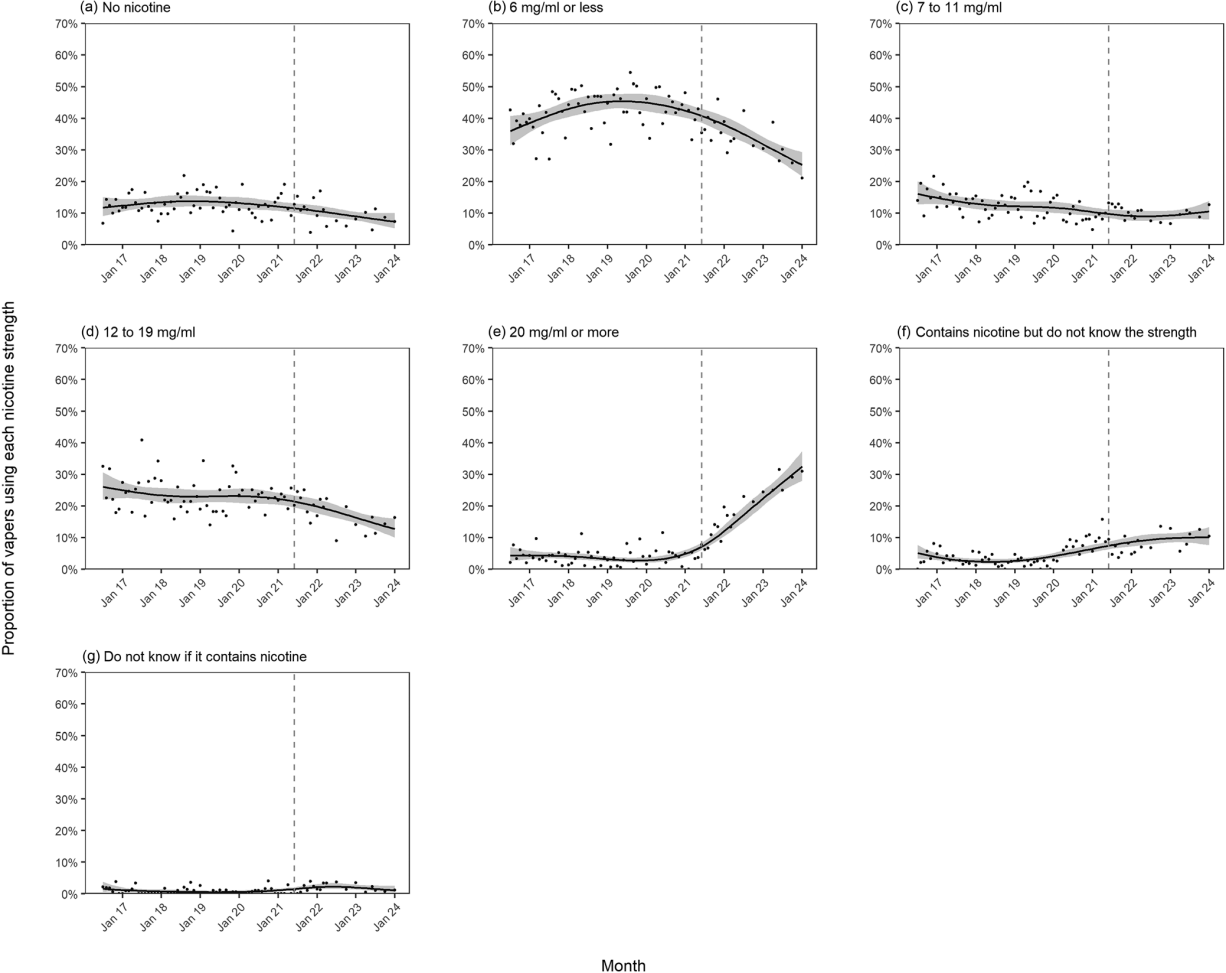
Trends in nicotine strengths of e‐liquids used by adult (≥18 years) vapers in England, July 2016 to January 2024. Unweighted sample size: *n* = 7314. Lines represent the modelled weighted proportion by monthly survey wave (modelled non‐linearly using restricted cubic splines with five knots). Shaded bands represent 95% CI. Points represent the unmodelled weighted proportion by month. The vertical dashed line indicates the timing of the start of the rise in popularity of disposable vaping in June 2021.

**FIGURE 2:**
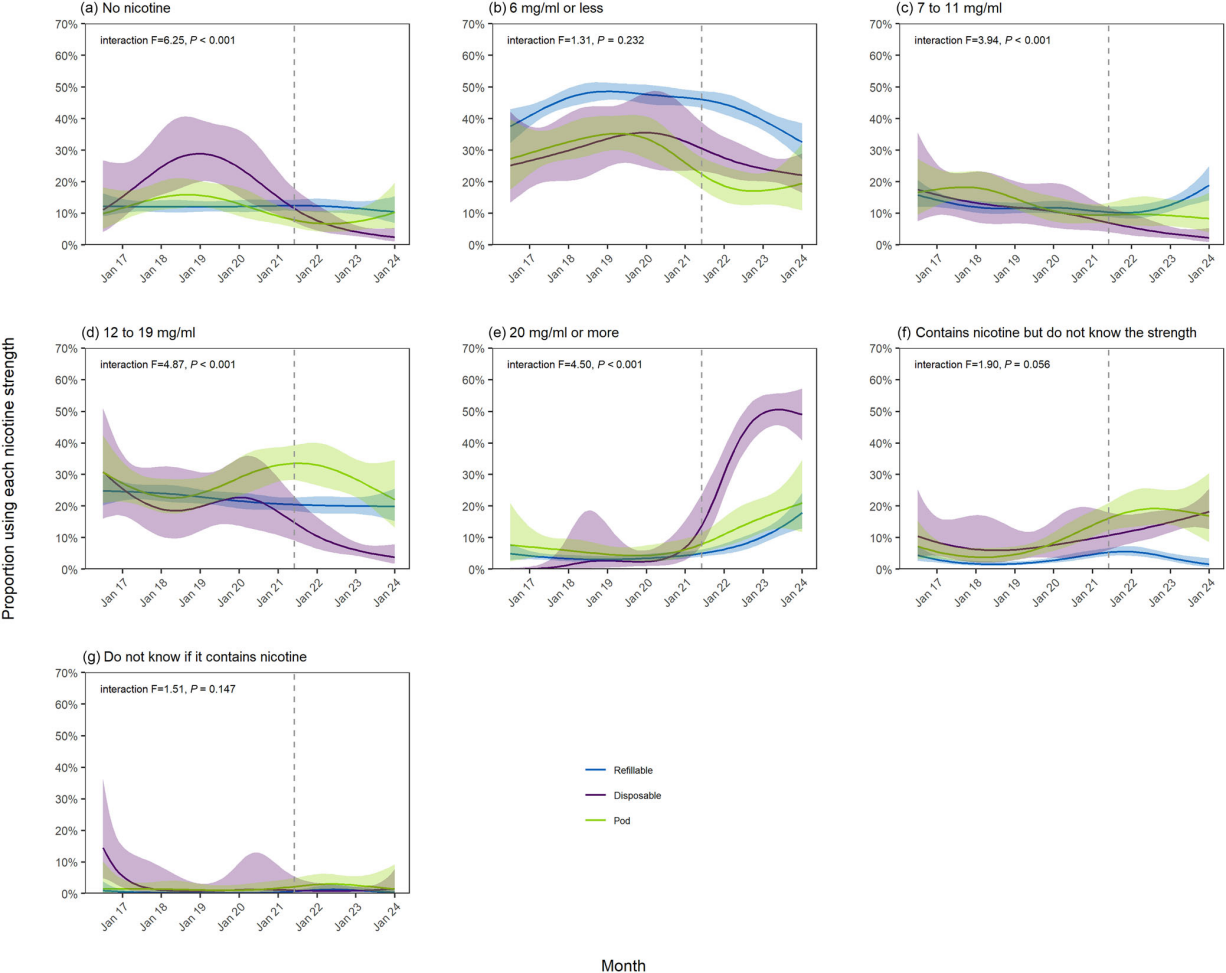
Trends in nicotine strengths of e‐liquids used by adult (≥18 years) vapers in England, July 2016 to January 2024, by the main device type used. Unweighted sample sizes: *n* = 5197 refillable; *n* = 880 disposable; *n* = 1131 pod. Lines represent the modelled weighted proportion by monthly survey wave (modelled non‐linearly using restricted cubic splines with five knots) and the main device type used. Shaded bands represent 95% CI. Points represent the unmodelled weighted proportion by month. The vertical dashed line indicates the timing of the start of the rise in popularity of disposable vaping in June 2021.

**FIGURE 3:**
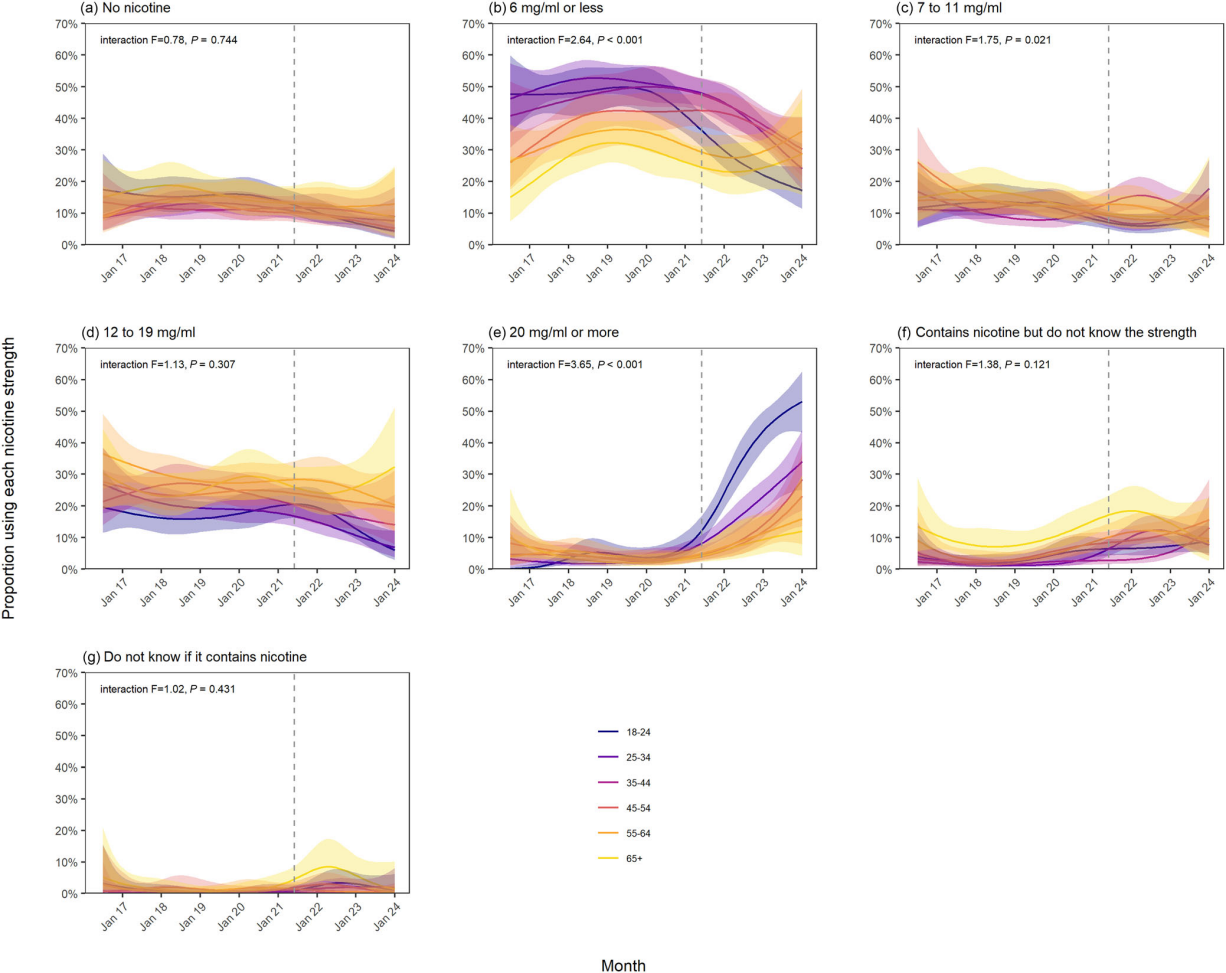
Trends in nicotine strengths of e‐liquids used by adult (≥18 years) vapers in England, July 2016 to January 2024, by age. Unweighted sample sizes: *n* = 1209 18‐ to 24‐year‐olds; *n* = 1650 25‐ to 34‐year‐olds; *n* = 1302 35‐ to 44‐year‐olds; *n* = 1337 45‐ to 54‐year‐olds; *n* = 1080 55‐ to 64‐year‐olds; *n* = 736 ≥65‐year‐olds. Lines represent the modelled weighted proportion by monthly survey wave (modelled non‐linearly using restricted cubic splines with five knots) and age. Shaded bands represent 95% CI. Points represent the unmodelled weighted proportion by month. The vertical dashed line indicates the timing of the start of the rise in popularity of disposable vaping in June 2021.

**FIGURE 4:**
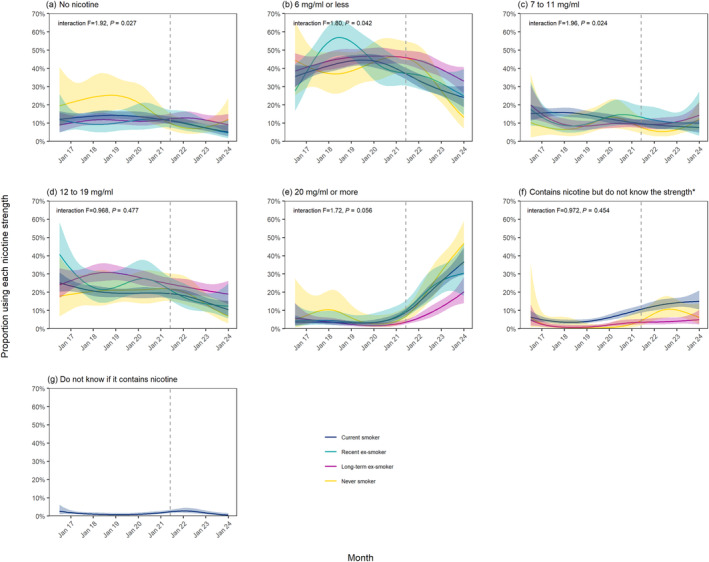
Trends in nicotine strengths of e‐liquids used by adult (≥18 years) vapers in England, July 2016 to January 2024, by smoking status. Unweighted sample sizes: *n* = 530 never smokers; *n* = 2251 long‐term (≥1 years) ex‐smokers; *n* = 624 recent (<1 year) ex‐smokers; *n* = 3909 current smokers. Lines represent the modelled weighted proportion by monthly survey wave (modelled non‐linearly using restricted cubic splines with five knots) and smoking status. Shaded bands represent 95% CI. Points represent the unmodelled weighted proportion by month. The vertical dashed line indicates the timing of the start of the rise in popularity of disposable vaping in June 2021. *Trends are not reported for recent ex‐smokers in (f) or for never, long‐term ex‐ or recent ex‐smokers in (g) because fewer than 30 participants in these groups endorsed these response options over the entire period, introducing substantial imprecision into the estimates.

Figure 1 shows modelled trends in nicotine strengths of e‐liquids used by vapers age ≥18 years in England between July 2016 and January 2024. Figures 2, 3 and 4 show trends by the main device type used, age and smoking status, respectively.

The proportion of vapers who reported using the highest‐strength (≥20 mg/mL) e‐liquids increased from an average of 3.8% (95% CI = 2.9%–5.0%) up to June 2021 to 32.5% (27.9%–37.4%) in January 2024 (Figure 1e). This was offset by declines over the same period in the proportion using nicotine‐free (from an average of 11.5% [10.2%–13.0%] up to June 2021 to 7.3% [5.2%–10.0%] in January 2024) (Figure 1a), ≤6 mg/mL (from 40.8% [38.6%–43.0%] to 25.3% [21.6%–29.3%], respectively) (Figure 1b) and 12–19 mg/mL e‐liquids (from 21.4% [19.6%–23.4%] to 12.7% [10.0–16.0%], respectively) (Figure 1d), whereas the proportion using 7–11 mg/mL e‐liquids remained relatively stable (at an average of 9.4% [7.9%–11.3%] between June 2021 and January 2024) (Figure 1c).

The proportion who said that the e‐cigarette they mainly used contained nicotine, but they did not know the strength increased in 2020 and 2021, from an average of 3.1% (2.4%–4.1%) up to December 2019 to 8.5% (7.2%–10.0%) by December 2021, then remained relatively stable at an average of 9.6% (8.1%–11.5%) between January 2022 and January 2024 (Figure (1f). The proportion who did not know if the e‐cigarette they mainly used contained nicotine was low (<2.5%) across the period (Figure 1g).

The overall increase in the proportion using ≥20 mg/mL e‐liquids since 2021 was observed across all device types, but was greatest among those mainly using disposables (increasing from an average of 2.6% [0.9%–9.3%] up to June 2021 to 49.0% [40.8%–57.2%] in January 2024, compared with 3.7% [2.8%–5.0%] to 17.9% [12.9–24.3%] and 5.7% [3.3%–9.8%] to 21.0% [11.8%–24.7%] among refillable and pod users, respectively) (Figure 2e).

It was also more pronounced among vapers ages 18 to 24 than those in older age groups, increasing from an average of 3.9% (2.1%–7.0%) up to June 2021 to 53.1% (43.3%–62.7%) in January 2024, compared with 3.9% (2.0%–7.6%) to 28.4% (18.7%–40.7%) among those ages 35 to 44 and 3.8% (1.9%–7.5%) to 12.1% (4.2%–29.8%) among those age ≥65 (age groups selected as examples) (Figure 3e). The decline in the use of ≤6 mg/mL e‐liquids was also greater in the youngest age group, falling from a high of 49.9% (43.8%–56.0%) in June 2019 to 17.2% (11.3%–25.2%) in January 2024, compared with 49.5% (44.1%–54.8%) to 30.2% (21.6%–40.5%) among those ages 35 to 44 and 32.0% (25.6%–39.2%) to 28.8% (15.8%–46.5%) among those age ≥65 over the same period (Figure 3b).

Although the increase in the proportion using ≥20 mg/mL e‐liquids since 2021 was observed across all smoking statuses, it was smallest among long‐term ex‐smokers. It increased from an average of 3.2% (1.9%–5.5%) up to June 2021 to 20.3% (14.0%–28.3%) in January 2024, compared with 6.1% (2.6%–14.6%) to 46.9% (34.9%–59.2%) among never smokers, 4.5% (2.1%–9.8%) to 30.5% (18.0%–46.7%) among recent ex‐smokers and 4.0% (2.8%–5.6%) to 36.8% (29.6%–44.5%) among current smokers (Figure 4e). The decline in the use of ≤6 mg/mL e‐liquids was greatest among never smokers, falling from 45.8% (37.1%–54.9%) in June 2021 to 13.1% (7.1%–23.0%) in January 2024, compared with 45.9% (42.1%–49.7%) to 33.0% (26.2%–40.7%) among long‐term ex‐smokers, 37.4% (30.6%–44.7%) to 24.9% (14.4%–39.6%) among recent ex‐smokers and 37.2% (34.1%–40.4%) to 24.1% (18.8%–30.3%) among current smokers (Figure 4b).

The overall increase in the proportion who said they did not know the strength of their nicotine‐containing e‐cigarette was observed across all smoking statuses, rising from 0.4% (0.1%–3.1%), 1.0% (0.4%–2.0%) and 4.0% (3.1%–5.2%) in January 2019 to 6.3% (2.7%–13.9%), 5.0% (2.4%–10.3%) and 15.1% (10.6%–21.0%) in January 2024 among never, long‐term ex‐ and current smokers, respectively (Figure 4f). However, the increase was limited to those who mainly used pod and disposable devices (reaching 16.9% [8.6%–30.5%] and 18.3% [12.7%–25.5%], respectively, by January 2024), with little overall change among those who mainly used refillables (1.5% [0.7%–3.5%] in January 2024) (Figure 2f).

### Nicotine strength of e‐liquids currently used by vapers in Great Britain

Table 1 provides data on the nicotine strength of e‐liquids used by vapers age ≥16 years in Great Britain in 2022 to 2024, overall and in relation to participant characteristics.

**TABLE 1:** Usual nicotine strength used by adult (≥16 years) vapers in Great Britain, January 2022–January 2024.

	*n* ^a^	Nicotine strength, % [95% CI]^b^						
		No nicotine	6 mg/mL or less	7–11 mg/mL	12–19 mg/mL	20 mg/mL or more	Do not know if it contains nicotine	Contains nicotine but do not know the strength
All adult vapers (≥16 y)	2373	8.7 [7.5–10.0]	30.5 [28.4–32.6]	9.1 [7.8–10.5]	15.3 [13.6–16.9]	25.0 [23.0–27.1]	1.8 [1.2–2.4]	9.5 [8.2–10.9]
Main device type								
Refillable	1267	11.8 [9.8–13.8]	39.0 [35.8–42.1]	13.2 [11.0–15.4]	20.2 [17.6–22.8]	11.5 [9.4–13.6]	0.9 [0.3–1.5]	3.5 [2.4–4.6]
Disposable	804	3.7 [2.4–5.0]	23.3 [20.0–26.6]	3.5 [2.1–5.0]	5.3 [3.5–7.1]	47.9 [44.0–51.8]	1.2 [0.3–2.1]	15.1 [12.4–17.8]
Pod	262	9.2 [5.5–13.0]	18.4 [13.1–23.6]	9.8 [5.6–13.9]	27.1 [21.3–33.0]	16.3 [11.2–21.4]	2.0 [0.4–3.7]	17.2 [11.6–22.7]
Vaping frequency								
Non‐daily	408	10.1 [6.9–13.3]	26.9 [21.9–32.0]	8.0 [4.9–11.2]	11.2 [7.9–14.5]	23.9 [19.1–28.6]	2.9 [0.9–4.8]	17.0 [12.8–21.1]
Daily	1613	7.9 [6.5–9.3]	31.9 [29.3–34.5]	9.8 [8.1–11.5]	16.1 [14.0–18.2]	25.2 [22.7–27.7]	1.5 [0.7–2.2]	7.6 [6.2–9.1]
Age (y)								
16–24	552	6.0 [4.0–8.0]	21.8 [17.8–25.7]	7.2 [4.9–9.6]	10.4 [7.3–13.5]	44.2 [39.5–48.8]	2.2 [0.7–3.8]	8.3 [5.8–10.7]
25–34	376	8.0 [5.5–10.6]	33.3 [28.8–37.8]	10.2 [7.3–13.1]	10.7 [7.8–13.6]	25.1 [20.9–29.3]	2.2 [0.7–3.7]	10.5 [7.5–13.4]
35–44	310	11.1 [7.5–14.7]	35.4 [30.0–40.8]	12.7 [8.9–16.6]	15.7 [11.6–19.8]	17.7 [13.5–21.9]	0.6 [0–1.3]	6.8 [4.1–9.5]
45–54	376	7.7 [4.9–10.5]	37.0 [31.4–42.6]	8.2 [4.9–11.6]	19.9 [15.3–24.5]	14.4 [9.9–18.8]	1.9 [0.4–3.4]	10.9 [7.0–14.9]
55–64	310	13.3 [8.9–17.6]	29.4 [23.6–35.1]	8.1 [5.0–11.3]	24.2 [18.8–29.6]	13.1 [8.3–17.9]	0.7 [0–1.6]	11.2 [7.2–15.2]
≥65	191	10.7 [5.7–15.8]	28.1 [20.4–35.7]	5.6 [1.9–9.3]	29.3 [21.5–37.1]	9.4 [4.4–14.3]	3.8 [0.9–6.6]	13.2 [8.1–18.3]
Gender^c^								
Men	1229	8.2 [6.5–9.8]	31.2 [28.2–34.2]	9.5 [7.6–11.4]	15.2 [13.0–17.5]	24.1 [21.3–27.0]	1.8 [1.0–2.7]	9.9 [7.9–11.9]
Women	1103	9.1 [7.2–11.0]	30.1 [27.0–33.2]	8.9 [7.0–10.9]	15.2 [12.7–17.6]	25.9 [22.9–28.9]	1.7 [0.8–2.6]	9.1 [7.3–10.9]
Occupational social grade								
ABC1 (more advantaged)	1407	8.5 [7.0–10.1]	30.8 [28.2–33.5]	8.9 [7.3–10.5]	15.0 [13.0–17.1]	25.3 [22.8–27.8]	1.3 [0.7–1.9]	10.1 [8.4–11.8]
C2DE (less advantaged)	966	8.9 [7.0–10.8]	30.2 [27.0–33.5]	9.3 [7.3–11.4]	15.5 [12.9–18.0]	24.8 [21.6–27.9]	2.3 [1.2–3.3]	9.1 [7.1–11.1]
Nation								
England	1791	8.4 [7.1–9.9]	30.5 [28.2–32.9]	9.2 [7.8–10.8]	15.0 [13.3–16.9]	25.5 [23.3–27.8]	1.9 [1.3–2.7]	9.5 [8.1–11.1]
Wales	186	14.2 [9.5–20.6]	27.0 [20.4–34.8]	10.0 [6.1–16.1]	17.0 [11.9–23.7]	23.5 [17.2–31.3]	2.1 [0.8–5.3]	6.2 [3.6–10.6]
Scotland	396	10 [7.1–13.9]	31.9 [27.1–37.0]	8.2 [5.6–11.8]	17.4 [13.7–21.8]	20.1 [16.1–24.9]	0.8 [0.3–2.2]	11.6 [8.4–15.8]
History of ≥1 diagnosed mental health conditions^d^								
No	737	9.1 [6.9–11.3]	32.9 [29.1–36.7]	8.3 [6.1–10.4]	14.5 [11.7–17.2]	21.6 [18.3–25.0]	2.9 [1.4–4.3]	10.8 [8.3–13.3]
Yes	744	8.2 [6.2–10.2]	32.4 [28.6–36.1]	9.0 [6.7–11.4]	17.2 [14.2–20.3]	25.1 [21.6–28.6]	1.6 [0.7–2.6]	6.4 [4.6–8.2]
Smoking status								
Long‐term (≥1 y) ex‐smoker	776	11.2 [8.7–13.7]	37.8 [33.8–41.7]	11.1 [8.5–13.8]	21.1 [17.7–24.6]	13.9 [10.9–16.8]	0.5 [0–1.2]	4.4 [2.6–6.1]
Recent (<1 y) ex‐smoker	223	8.3 [4.7–11.9]	31.8 [24.7–38.9]	9.0 [4.5–13.5]	13.5 [8.5–18.5]	27.4 [20.4–34.4]	3.3 [0.4–6.1]	6.7 [2.9–10.4]
Current smoker	1078	7.0 [5.4–8.7]	27.4 [24.3–30.5]	8.1 [6.2–10.1]	13.1 [10.9–15.3]	28.8 [25.6–31.9]	1.9 [1.0–2.8]	13.7 [11.4–16.0]
Never smoker	296	9.1 [5.4–12.9]	23.6 [17.9–29.2]	7.9 [4.7–11.2]	10.5 [6.3–14.6]	36.0 [29.8–42.2]	3.4 [1.0–5.9]	9.4 [5.8–13.1]
Strength of urges to smoke^e^								
Not at all	237	6.7 [3.6–9.8]	31.2 [24.4–38.0]	11.7 [6.6–16.8]	11.5 [7.1–15.8]	25.8 [19.1–32.5]	1.3 [0–3.4]	11.7 [6.8–16.7]
Slight	251	7.0 [3.7–10.2]	30.2 [23.5–36.8]	8.2 [4.5–11.9]	9.1 [5.2–13.0]	30.2 [23.8–36.6]	2.3 [0.2–4.5]	13.0 [8.3–17.8]
Moderate	461	7.2 [4.6–9.7]	26.9 [22.2–31.5]	8.4 [5.4–11.3]	14.4 [10.8–18.1]	27.0 [22.2–31.8]	1.5 [0.4–2.6]	14.8 [11.1–18.5]
Strong	212	7.2 [2.9–11.4]	28.0 [20.9–35.1]	6.4 [2.1–10.7]	16.4 [10.9–21.9]	31.3 [24.1–38.4]	1.4 [0–3.1]	9.3 [5.3–13.3]
Very strong	60	5.1 [0–11.5]	29.7 [15.8–43.7]	6.8 [0.5–13.1]	16.4 [6.1–26.8]	32.8 [18.4–47.1]	0 [0–0]	9.1 [1.6–16.7]
Extremely strong	42	13.4 [2.3–24.6]	25.9 [9.8–42.1]	5.6 [0–13.6]	14.2 [3.0–25.4]	35.2 [17.2–53.1]	1.5 [0–4.5]	4.1 [0–9.4]

^a^ Unweighted sample size. Note that there were some missing data on main device type (*n* = 40), age (*n* = 1), gender (*n* = 41, including those who identified as non‐binary), and history of mental health conditions (*n* = 892, because data were not collected after June 2023 and were only collected from ~50% of participants surveyed in Wales and Scotland), so numbers within subgroups do not sum to the total.

^b^ Weighted row percentages.

^c^ Participants who described their gender in another way were excluded from analyses by gender because of low numbers.

^d^ Mental health conditions were not assessed after June 2023, so results are based on aggregated data across January 2022–June 2023.

^e^ Self‐reported ratings of the strength of urges to smoke in the past 24 hours among past‐year smokers.

Overall, 89.5% (95% CI = 88.1%–90.8%) said they usually used e‐cigarettes containing nicotine, 8.7% (7.5%–10.0%) said they used nicotine‐free cigarettes and 1.8% (1.2%–2.4%) were unsure. The most commonly used nicotine strength was ≤6 mg/mL (30.5% of vapers), followed by ≥20 mg/mL (25.0%), 12–19 mg/mL (15.3%) and 7–11 mg/mL (9.1%).

However, the nicotine strength of e‐liquids used differed across subgroups of vapers. There were clear differences between those using different e‐cigarette device types. Almost half (47.9%) of disposable users reported using ≥20 mg/mL e‐liquids compared with 16.3% of pod users and 11.5% of refillable users. By contrast, 50.8% of refillable users reported using ≤6 mg/mL or nicotine‐free e‐liquids, compared with 27.0% of disposable users and 27.6% of pod users. Refillable users were least likely to say that they did not know the strength of their nicotine‐containing e‐cigarette (3.5% vs 15.1% and 17.2% of disposable and pod users, respectively).

Those vaping non‐daily were more likely than daily users to say that they did not know the strength of their nicotine‐containing e‐cigarette (17.0% vs 7.6%, respectively), but use of the highest strength (≥20 mg/mL) e‐liquids was similar among non‐daily and daily vapers (23.9% vs 25.2%, respectively).

There were also differences by age, with almost half (44.2%) of 16 to 24‐year‐olds using the highest strength (≥20 mg/mL) e‐liquids, compared with 9.4% to 25.1% among older age groups. This age difference was observed across users of refillable (Table S3), disposable (Table S4) and pod (Table S5) devices.

Finally, there were differences by smoking status. Never smokers (36.0%), current smokers (28.8%) and recent ex‐smokers (27.4%) were more likely than long‐term ex‐smokers (13.9%) to report using the highest strength (≥20 mg/mL) e‐liquids. They were also much more likely to say that they did not know whether their device contained nicotine (3.4%, 1.9% and 3.3% vs 0.5%, respectively) or that they did not know the strength of their nicotine‐containing e‐cigarette (9.4%, 13.7% and 6.7% vs 4.4%, respectively). The majority (87.4%) of never smokers were using nicotine‐containing e‐cigarettes, most commonly with ≥20 mg/mL e‐liquids (36.0%). Of those using higher‐strength e‐liquids (≥12 mg/mL), never, recent ex‐ and current smokers were more likely to be using ≥20 mg/mL than 12–19 mg/mL, but the opposite was true for long‐term ex‐smokers. A higher proportion of long‐term ex‐smokers than current and never smokers reported using low‐nicotine (≤6 mg/mL) e‐liquids (37.8% vs 27.4% and 23.6%, respectively).

There were no notable differences in nicotine strength by gender, occupational social grade, history of mental health conditions or (among smokers) level of cigarette dependence.

### Groups that would currently be most affected by the proposed Vaping Products Duty structure

Table 2 shows the results of the multinomial logistic regression models, which summarise subgroup differences in the use of e‐liquids that would be taxed at intermediate and higher levels, compared with lower levels, according to the proposed Vaping Products Duty structure.

**TABLE 2:** Associations between usual nicotine strength and user characteristics among adult (≥16 years) vapers in Great Britain, January 2022–January 2024.

	*n* ^a^	Nicotine strength vs no nicotine, OR [95% CI]^b^	
		11 mg/mL or less	12 mg/mL or more
Main device type			
Refillable	1207	Ref	Ref
Disposable	666	1.62 [1.10–2.38]	5.21 [3.57–7.60]
Pod	213	0.69 [0.44–1.09]	1.75 [1.13–2.73]
Vaping frequency			
Non‐daily	327	Ref	Ref
Daily	1465	1.53 [1.06–2.19]	1.51 [1.05–2.16]
Age (y)			
16–24	496	Ref	Ref
25–34	481	1.12 [0.73–1.72]	0.49 [0.32–0.75]
35–44	364	0.90 [0.58–1.39]	0.33 [0.21–0.52]
45–54	333	1.21 [0.73–2.02]	0.49 [0.29–0.81]
55–64	272	0.58 [0.36–0.96]	0.31 [0.19–0.50]
≥65	154	0.65 [0.34–1.23]	0.39 [0.21–0.73]
Gender^c^			
Men	1095	Ref	Ref
Women	971	0.86 [0.65–1.14]	0.94 [0.71–1.24]
Occupational social grade			
ABC1 (more advantaged)	1251	Ref	Ref
C2DE (less advantaged)	850	0.96 [0.72–1.26]	0.96 [0.72–1.27]
Occupational social grade			
England	1582	Ref	Ref
Wales	167	0.55 [0.30–1.03]	0.61 [0.33–1.13]
Scotland	352	0.85 [0.51–1.41]	0.79 [0.47–1.33]
History of ≥1 diagnosed mental health conditions^d^			
No	640	Ref	Ref
Yes	677	1.10 [0.77–1.59]	1.28 [0.89–1.85]
Smoking status			
Long‐term (≥1 y) ex‐smoker	741	Ref	Ref
Recent (<1 y) ex‐smoker	203	1.13 [0.68–1.87]	1.61 [0.96–2.67]
Current smoker	898	1.15 [0.84–1.58]	1.93 [1.40–2.66]
Never smoker	259	0.79 [0.51–1.22]	1.59 [1.04–2.45]
Strength of urges to smoke^e^			
Not at all	208	Ref	Ref
Slight	212	0.86 [0.44–1.69]	1.02 [0.52–2.02]
Moderate	385	0.77 [0.43–1.39]	1.06 [0.59–1.91]
Strong	184	0.75 [0.37–1.51]	1.20 [0.60–2.42]
Very strong	53	1.12 [0.34–3.75]	1.78 [0.54–5.89]
Extremely strong	38	0.36 [0.12–1.06]	0.64 [0.23–1.79]

^a^ Unweighted sample size. Note these numbers differ from those in Table 1 because those who responded that they did not know the nicotine strength were excluded.

^b^ OR calculated using multinomial logistic regression (with no nicotine as the reference category), univariate models adjusted for survey wave.

^c^ Participants who described their gender in another way were excluded from analyses by gender because of low numbers.

^d^ Mental health conditions were not collected after June 2023, so results are based on data collected between January 2022 and June 2023.

^e^ Self‐reported ratings of the strength of urges to smoke in the past 24 hours among past‐year smokers.

There were significant differences by the main device type used, vaping frequency, age and smoking status. Relative to those who mainly used refillable devices, disposable users had 1.62 times higher odds of using mid‐strength (≤11 mg/mL) e‐liquids and 5.21 times higher odds of using high‐strength (≥12 mg/mL) e‐liquids than nicotine‐free e‐liquids and pod users had 1.75 times higher odds of using high‐strength e‐liquids.

Relative to those who vaped non‐daily, daily users had 1.53 and 1.51 times higher odds of using mid‐ and high‐strength e‐liquids than nicotine‐free e‐liquids, respectively.

The odds of using high‐strength e‐liquids versus nicotine‐free e‐liquids were lower among older (≥25y) age groups than those ages 16 to 24 years (OR range = 0.31–0.49), but the odds of using mid‐strength e‐liquids were similar across age groups.

Relative to long‐term ex‐smokers, current smokers had 93% higher odds of using high‐strength versus nicotine‐free e‐liquids, recent ex‐smokers had 61% higher odds and never smokers had 59% higher odds. The odds of using mid‐strength e‐liquids were more similar across smoking statuses.

There were no notable differences across other subgroups.

## DISCUSSION

This study provides a comprehensive picture of the nicotine strengths of e‐liquids used by adult vapers in Great Britain, with five key findings.

First, although nine in 10 vapers reported using e‐cigarettes that contain nicotine, there have been notable changes in the strengths used in England since 2016. In particular, there has been a sharp rise in the proportion of vapers using the highest‐strength (≥20 mg/mL) e‐liquids since disposable e‐cigarettes started to become popular in the spring of 2021 [2, 26], offset by a decline in the proportion using lower‐strength e‐liquids (particularly ≤6 mg/mL). For the majority of the time the question was assessed, the question did not distinguish between 20 mg/mL (the legal limit) and higher concentrations. In the most recent data, the question distinguished between the two, and indicated that more than 90% of this group use the legal limit rather than stronger concentrations. Although the increase in use of high‐strength nicotine e‐liquids was particularly pronounced among those using disposables, it was also observed across users of refillable and pod devices. It was greatest among 18 to 24‐year‐olds, consistent with the rise in use of disposable e‐cigarettes being greatest at younger ages [2, 26], and was similarly pronounced in people of all smoking statuses (including never smokers), except long‐term ex‐smokers.

Second, there has been an increase since 2020 in the proportion of vapers using disposable and pod devices who did not know how strong their nicotine‐containing e‐liquid was. It is possible that this increase is because of changes in where people are buying their vaping products since the Covid‐19 pandemic. Before the pandemic, most vapers said that they usually bought their e‐cigarettes and e‐liquids from specialist vape shops [27], where staff are knowledgeable about the products and offer advice on nicotine strength [28]. However, vape shops were forced to close during periods of lockdown [29], which saw a shift toward online purchasing [27]. In addition, since disposable e‐cigarettes were introduced to the market in 2021, supermarkets and convenience stores have become the most popular source of purchase [27]. Typically, devices in these locations are stored behind a counter and people cannot easily browse or inspect products before stating which device they would like to purchase. Better labelling and display by nicotine strength may be required to make the nicotine strength of products sold in these outlets clearer to consumers. If people are using illegal products, they may not clearly display nicotine content.

Third, the nicotine strength of e‐liquids used currently tended to be higher among those who mainly use disposable devices. Although the UK government plans to ban disposable e‐cigarettes [8], it is unlikely that this will result in a return to the profile of nicotine strength use before disposables became popular in 2021. Manufacturers are already responding rapidly to an impending ban by introducing reusable (rechargeable and refillable) models to the market that are very similar in design and price to popular disposable models—including the nicotine strength of the e‐liquids they contain. In addition, new e‐cigarettes (whether disposable or not) typically use nicotine salts e‐liquids, which allow for higher nicotine strengths to be inhaled without the harshness people experience when they use high‐strength e‐liquids containing freebase nicotine [6].

Fourth, the nicotine strengths used also tended to be higher among those ages 16 to 24 years (whether or not they are using disposables), and in people of all smoking statuses (including never smokers) except long‐term ex‐smokers. This suggests that the proposed Vaping Products Duty would disproportionately affect young vapers who have never smoked and may, therefore, contribute to reducing uptake (i.e. progression to regular use from experimentation) in this group, a stated policy objective. However, our results suggest it is not just never smokers who would be affected, but also current and recent ex‐smokers who also tend to use higher nicotine strengths compared with long‐term ex‐smokers. This could have a number of unintended consequences.

If the duty discourages smokers from trying to quit with e‐cigarettes or prompts them to use lower‐strength e‐liquids, it could undermine quitting and perpetuate smoking. Comparisons of the effectiveness of different doses of nicotine in e‐cigarettes are limited [13]. One RCT (conducted in the United States where higher nicotine strengths are permitted than in the United Kingdom) found quit rates were 2.5 times higher among smokers randomised to receive an e‐cigarette containing 36 mg/mL e‐liquid than those who received 8 mg/mL, but the 95% CI included no difference (RR = 2.50 [95% CI = 0.80–7.77]) [14]. We found that although current smokers tended to use higher‐strength e‐liquids than long‐term ex‐smokers, one in three reported using low‐strength (<6 mg/mL) or nicotine‐free e‐cigarettes. Given higher‐strength e‐liquids are more effective in relieving cravings for tobacco [12], people who want to use e‐cigarettes to quit smoking could be encouraged to use higher‐strength products (at least in the short term) to potentially increase their chances of quitting [14]. However, the structure of the proposed duty will make it more expensive for smokers who do so. Our data do not tell us about the nicotine strength of e‐liquids used by smokers in quit attempts, which may be higher than the average among current smokers and recent ex‐smokers (as those who quit may reduce the nicotine strength used gradually after quitting).

If the duty encourages ex‐smokers who vape to stop vaping or to switch to lower‐strength e‐liquids, there is a risk it could trigger relapse to smoking (although there is little direct evidence of the impact of e‐cigarettes on long‐term relapse, and people have also speculated that long‐term nicotine dependence may be a greater risk factor for long‐term relapse). This seems unlikely for long‐term ex‐smokers who reported using the lowest nicotine strengths, which may reflect people ‘tapering down’ their nicotine use over time or having quit with and continued using older‐generation refillable devices (which, as we found, are typically used with lower‐strength e‐liquids than modern disposables). If it does not affect the risk of long‐term relapse to smoking, then there are likely to be health benefits because vaping long‐term is not harmless [16]. However, higher strengths may be important for recent ex‐smokers (who tended to use these), who may benefit from using high‐strength nicotine e‐liquid in the early phases of switching to minimise the risk of relapse [30].

There is also a risk that the duty could worsen misperceptions about the harms of vaping. Although we did not analyse perceptions in this study, recent data show smokers' perceptions of the relative harms of e‐cigarettes compared with cigarettes are as bad as they have ever been, with more than half believing they are equally or more harmful [31]. Many people misattribute the cause of smoking‐related disease to nicotine [32, 33]. Applying higher duty rates to higher‐strength nicotine products may have the unwanted effect of worsening or maintaining these misperceptions if people think the tax is because the harms of these products are comparable to smoking rather than to reduce youth use.

The fifth key finding was that nicotine strength preferences did not differ substantially according to markers of disadvantage (e.g. by occupational social grade or history of mental health conditions) or by level of cigarette dependence among vapers who smoked. Although this provides some reassurance that levying higher rates of tax on higher‐strength e‐liquids may not disproportionately affect disadvantaged or more dependent smokers who vape, our data only reflect vapers' current nicotine strength preferences. They do not offer insight into how vapers' choice of nicotine strength may change when the duty is introduced.

If any of the potential responses outlined above are greater among disadvantaged groups, it could have a negative equity impact. E‐cigarettes are an important intervention for reducing smoking‐related inequalities, because they offer a less harmful way of using nicotine [16] without the need to quit nicotine altogether, which can be appealing for people with difficult lives who are not ready to consider total nicotine abstinence. Vaping is also cheaper than smoking [34] and price is a motivator for those on low incomes. The UK government's ‘Swap to Stop’ initiative to provide a million free e‐cigarette starter packs (alongside behavioural support to quit) is focused on reducing inequalities [35]. The Vaping Products Duty will need to be carefully communicated so as not to dissuade people who could benefit most from taking up the offer of a free e‐cigarette starter pack from their local stop smoking service.

Further research is urgently needed to understand the extent to which these potential unintended consequences are likely to occur and how they can be mitigated. In addition, more research is needed into vapers who have never regularly smoked, who increasingly reported using high‐strength nicotine e‐liquids. Although there is no evidence that the increase in use of higher‐strength e‐liquids has resulted in higher smoking rates, and previous research also has not found evidence of a gateway from vaping to smoking at the population level [36, 37], the increase in use of high‐strength e‐liquids among never smokers needs to be monitored in terms of possible gateway effects in the future.

Strengths of the study include the representative sample and up‐to‐date data on nicotine strength preferences. There were also limitations. All data were self‐reported. Nicotine strength was not assessed in every wave in 2022 and 2023, meaning there were some missing data at the individual level. However, the use of splines effectively interpolated at the aggregate level using information before and after the missing time points to model the trends across the period. Data were not collected in Wales and Scotland before October 2020, so our analyses of time trends were restricted to vapers in England. Sample sizes within some subgroups were relatively small, which meant estimates were imprecise (as indicated by wide 95% CIs), and there may be differences in nicotine strength preferences between groups that we did not detect. In addition, the 20 mg/mL nicotine strength classification included e‐liquids at and exceeding the maximum legal limit in Great Britain [16], therefore, we were unable to analyse trends in the use of nicotine strengths that exceed the legal limit. We did not collect data on the amount of e‐liquid used, which will be a key influence on expenditure. Finally, we did not differentiate between reasons for use, so our data do not offer insight into nicotine strengths used by people vaping for the purpose of quitting, cutting down, or for other reasons.

In conclusion, use of high‐strength nicotine e‐liquids in England has increased sharply since disposable e‐cigarettes have become popular. Most adult vapers in Great Britain use e‐cigarettes that contain nicotine but different subgroups use different nicotine strengths with the strength tending to be higher among those who mainly use disposable devices, those aged 16 to 24 years and lower among long‐term ex‐smokers. In applying higher rates of tax to higher‐strength nicotine e‐liquids, the proposed Vaping Products Duty may be effective in reducing progression from experimentation to regular use and dependence among young adults (and potentially youth, who were not assessed here), including those who have never smoked. There may, however, be implications arising from the proposed duty for smokers trying to quit by vaping, which need taking into account before finalising the tax structure. Monitoring the outcomes and any unintended consequences from the policy will be important.

## AUTHOR CONTRIBUTIONS

**Sarah Jackson:** Conceptualization (equal); formal analysis (lead); investigation (equal); methodology (equal); visualization (lead); writing—original draft (lead); writing—review and editing (equal). **Jamie Brown:** Data curation (lead); funding acquisition (equal); investigation (equal); methodology (equal); writing—review and editing (equal). **Lion Shahab:** Funding acquisition (equal); investigation (equal); methodology (equal); writing—review and editing (equal). **Deborah Arnott:** Investigation (equal); writing—review and editing (equal). **Linda Bauld:** Investigation (equal); writing—review and editing (equal). **Sharon Cox:** Conceptualization (equal); investigation (equal); methodology (equal); writing—review and editing (equal).

## ACKNOWLEDGEMENTS

None.

## DECLARATION OF INTERESTS

J.B. has received unrestricted research funding from Pfizer and J&J, who manufacture smoking cessation medications. L.S. has received honoraria for talks, unrestricted research grants and travel expenses to attend meetings and workshops from manufactures of smoking cessation medications (Pfizer; J&J), and has acted as paid reviewer for grant awarding bodies and as a paid consultant for health care companies. All authors declare that they have never had any financial links with tobacco companies, e‐cigarette manufacturers, or their representatives.

## Supporting information


**Table S1.** Weighted sample characteristics.
**Table S2.** Weighted vaping characteristics and age by smoking status.
**Table S3.** Usual nicotine strength used by adult (≥16y) vapers in Great Britain, January 2022–‐January 2024 – refillable device users.
**Table S4.** Usual nicotine strength used by adult (≥16y) vapers in Great Britain, January 2022–‐January 2024 – disposable device users.
**Table S5.** Usual nicotine strength used by adult (≥16y) vapers in Great Britain, January 2022–‐January 2024 – pod device users.

## Data Availability

The data used for these analyses are available on Open Science Framework.
