# Supplementary material for: Nicotine strength of e‐liquids used by adult vapers in Great Britain: A population survey 2016 to 2024
Source: Addiction. 2024 Jun 19;120(3):468–82. doi: 10.1111/add.16576 (PMC11813722; doi:10.1111/add.16576)
Supplement: Supplementary file 1 — Table S1. Weighted sample characteristics. Table S2. Weighted vaping characteristics and age by smoking status. Table S3. Usual nicotine strength used by adult (≥16y) vapers in Great Britain, January 2022–‐January 2024 – refillable device users. Table S4. Usual nicotine strength used by adult (≥16y) vapers in Great Britain, January 2022–‐January 2024 – disposable device users. Table S5. Usual nicotine strength used by adult (≥16y) vapers in Great Britain, January 2022–‐January 2024 – pod device users. [file ADD-120-468-s001.docx]

##### Table S1. Weighted sample characteristics

|  | **Trend analyses:**  **adults aged ≥18 years in England**  **(*n*^1^=7,314)** | **Descriptive analyses (2022-24): adults aged ≥16 years in Great Britain**  **(*n*^1^=2,373)** |
| --- | --- | --- |
|  |  |  |
| Main device type |  |  |
| Refillable | 72.7 | 52.1 |
| Disposable | 12.4 | 36.7 |
| Pod | 15.0 | 11.3 |
| *Missing* | *106* | *40* |
|  |  |  |
| Vaping frequency^2^ |  |  |
| Non-daily | 23.3 | 21.1 |
| Daily | 76.7 | 78.9 |
| *Missing* | *820* | *352* |
|  |  |  |
| Age (years) |  |  |
| Mean (SD) | 40.8 (15.2) | 37.4 (15.3) |
| 16-24^3^ | 16.2 | 25.7 |
| 25-34 | 25.4 | 26.1 |
| 35-44 | 19.7 | 18.1 |
| 45-54 | 18.4 | 14.1 |
| 55-64 | 12.3 | 10.3 |
| ≥65 | 7.9 | 5.7 |
| *Missing* | *-* | *1* |
|  |  |  |
| Gender^4^ |  |  |
| Men | 54.7 | 51.7 |
| Women | 44.8 | 47.3 |
| Other | 0.5 | 1.0 |
| *Missing* | *7* | *14* |
|  |  |  |
| Occupational social grade |  |  |
| ABC1 (more advantaged) | 46.0 | 45.9 |
| C2DE (less advantaged) | 54.0 | 54.1 |
|  |  |  |
| Nation |  |  |
| England | 100.0 | 89.1 |
| Wales | - | 3.7 |
| Scotland | - | 7.2 |
|  |  |  |
| History of ≥1 diagnosed mental health conditions^5^ |  |  |
| No | - | 48.2 |
| Yes | - | 51.8 |
| *Missing* | *-* | *892^4^* |
|  |  |  |
| Smoking status |  |  |
| Long-term (≥1y) ex-smoker | 30.8 | 31.1 |
| Recent (<1y) ex-smoker | 8.8 | 9.7 |
| Current smoker | 53.1 | 45.3 |
| Never smoker | 7.3 | 13.9 |
|  |  |  |
| Strength of urges to smoke^6^ |  |  |
| Not at all | 9.4 | 11.6 |
| Slight | 16.5 | 19.6 |
| Moderate | 47.2 | 41.1 |
| Strong | 18.8 | 19.0 |
| Very strong | 5.5 | 5.5 |
| Extremely strong | 2.7 | 3.3 |
| *Missing* | *49* | *31* |
|  |  |  |

^1^ Unweighted sample size.

^2^ Vaping frequency was assessed with multiple response options ranging from ‘not every day and less than once a week’ to ‘12+’ times a day. All missing cases were participants who responded that they did not know how often they vaped.

^3^ Age 18-24 for trend analyses.

^4^ Participants who described their gender in another way were excluded from analyses by gender due to low numbers.

^5^ Mental health conditions were not assessed after June 2023, so results are based on aggregated data across January 2022 – June 2023. This variable was only collected in ~50% of participants surveyed in Wales and Scotland (the number of missing cases includes those not asked). Survey weights are applied to account for this in our analyses. Data on mental health are not presented for the trend analysis sample as this variable was not assessed across the whole period and is not included in these analyses.

^6^ Self-reported ratings of the strength of urges to smoke in the past 24 hours among past-year smokers.

Note: Data are shown as weighted column percentages, unless otherwise specified. There were some missing data (unweighted *n*s indicated in the table); valid percentages are shown for ease of interpretation.

##### Table S2. Weighted vaping characteristics and age by smoking status

|  | **Trend analyses:**  **adults aged ≥18 years in England** | | | |  | **Descriptive analyses (2022-24): adults aged ≥16 years in Great Britain** | | | |
| --- | --- | --- | --- | --- | --- | --- | --- | --- | --- |
|  | **Never smoker** | **Long-term ex-smoker** | **Recent ex-smoker** | **Current smoker** |  | **Never smoker** | **Long-term ex-smoker** | **Recent ex-smoker** | **Current smoker** |
|  |  |  |  |  |  |  |  |  |  |
| Unweighted *N* | 530 | 2251 | 624 | 3909 |  | 296 | 776 | 223 | 1078 |
|  |  |  |  |  |  |  |  |  |  |
| Main device type |  |  |  |  |  |  |  |  |  |
| Refillable | 65.7 | 82.8 | 70.7 | 68.0 |  | 41.6 | 74.3 | 47.3 | 40.8 |
| Disposable | 23.2 | 5.3 | 14.5 | 14.6 |  | 50.3 | 15.4 | 41.8 | 46.2 |
| Pod | 11.1 | 11.9 | 14.8 | 17.3 |  | 8.1 | 10.3 | 10.8 | 13.0 |
|  |  |  |  |  |  |  |  |  |  |
| Vaping frequency |  |  |  |  |  |  |  |  |  |
| Non-daily | 21.8 | 9.2 | 11.8 | 33.0 |  | 27.0 | 11.6 | 10.0 | 27.8 |
| Daily | 78.2 | 90.8 | 88.2 | 67.0 |  | 73.0 | 88.4 | 90.0 | 72.2 |
|  |  |  |  |  |  |  |  |  |  |
| Age (years) |  |  |  |  |  |  |  |  |  |
| Mean (SD) | 34.4 (14.7) | 44.9 (14.5) | 37.6 (13.9) | 39.8 (15.3) |  | 28.2 (12.7) | 43.1 (14.6) | 35.6 (13.5) | 36.7 (15.2) |
| 16-24^3^ | 35.1 | 6.5 | 18.9 | 18.8 |  | 57.3 | 9.5 | 24.6 | 27.4 |
| 25-34 | 24.0 | 22.2 | 31.7 | 26.4 |  | 19.8 | 24.5 | 33.4 | 27.5 |
| 35-44 | 16.4 | 23.2 | 19.9 | 18.1 |  | 11.0 | 23.7 | 20.4 | 16.0 |
| 45-54 | 13.9 | 21.5 | 16.4 | 17.6 |  | 6.6 | 18.8 | 10.3 | 14.0 |
| 55-64 | 6.6 | 15.9 | 8.3 | 11.7 |  | 3.6 | 14.4 | 7.2 | 10.2 |
| ≥65 | 4.1 | 10.7 | 4.7 | 7.4 |  | 1.7 | 9.1 | 4.2 | 4.9 |
|  |  |  |  |  |  |  |  |  |  |

^1^ Unweighted sample size.

Note: Data are shown as weighted column percentages, unless otherwise specified. There were some missing data (see Table S1); valid percentages are shown for ease of interpretation.

##### Table S2. Usual nicotine strength used by adult (≥16y) vapers in Great Britain, January 2022-January 2024 – refillable device users

|  |  | **Nicotine strength, % [95% CI]^2^** | | | | | | |
| --- | --- | --- | --- | --- | --- | --- | --- | --- |
|  | ***N*^1^** | **No nicotine** | **6 mg/ml or less** | **7 to 11 mg/ml** | **12 to 19 mg/ml** | **20 mg/ml or more** | **Do not know if it contains nicotine** | **Contains nicotine but do not know the strength** |
|  |  |  |  |  |  |  |  |  |
| All adult vapers (≥16y) | 1267 | 11.8 [9.8–13.8] | 39 [35.8–42.1] | 13.2 [11.0–15.4] | 20.2 [17.6–22.8] | 11.5 [9.4–13.6] | 0.9 [0.3–1.5] | 3.5 [2.4–4.6] |
|  |  |  |  |  |  |  |  |  |
| Vaping frequency |  |  |  |  |  |  |  |  |
| Non-daily | 153 | 14.9 [8.7–21.2] | 38.3 [28.9–47.6] | 14.4 [7.6–21.1] | 15.4 [8.8–22.0] | 12.1 [6.2–17.9] | 0.6 [0–1.8] | 4.4 [0.8–7.9] |
| Daily | 901 | 11.1 [8.8–13.4] | 40.5 [36.8–44.3] | 13.7 [11.0–16.3] | 20.0 [16.9–23.1] | 10.5 [8.1–12.9] | 1.0 [0.2–1.7] | 3.2 [2.0–4.5] |
|  |  |  |  |  |  |  |  |  |
| Age (years) |  |  |  |  |  |  |  |  |
| 16-24 | 159 | 15.3 [9.3–21.2] | 25.6 [17.7–33.6] | 15.3 [9.1–21.4] | 20.2 [12.5–27.9] | 20.4 [13.3–27.4] | 1.6 [0–4.0] | 1.7 [0–4.0] |
| 25-34 | 284 | 10.9 [6.8–15.0] | 43.1 [36.4–49.7] | 15.1 [10.3–20.0] | 12.7 [8.2–17.2] | 13.5 [9.0–17.9] | 1.5 [0–3.1] | 3.2 [0.8–5.6] |
| 35-44 | 226 | 12.3 [7.2–17.3] | 43.6 [36.2–51.0] | 15.2 [9.9–20.6] | 19.2 [13.2–25.2] | 8.8 [4.6–13.1] | 0 [0–0] | 0.9 [0–1.9] |
| 45-54 | 253 | 7.7 [4.2–11.2] | 44.8 [37.6–52.0] | 10.4 [5.6–15.1] | 22.9 [16.8–29.0] | 7.5 [3.3–11.8] | 0.8 [0–1.9] | 5.9 [2.3–9.4] |
| 55-64 | 219 | 12.2 [7.3–17.1] | 35.6 [28.3–42.9] | 10.9 [6.5–15.3] | 27.6 [20.8–34.5] | 8.9 [4.0–13.8] | 0.5 [0–1.5] | 4.3 [1.7–6.9] |
| ≥65 | 125 | 15.0 [7.5–22.5] | 30.0 [20.5–39.6] | 7.4 [2.0–12.8] | 29.8 [20.1–39.6] | 8.8 [2.3–15.4] | 0.7 [0–2.0] | 8.2 [3.2–13.3] |
|  |  |  |  |  |  |  |  |  |
| Gender^3^ |  |  |  |  |  |  |  |  |
| Men | 712 | 11.8 [9.1–14.4] | 39.0 [34.8–43.2] | 12.6 [9.7–15.4] | 19.1 [15.8–22.5] | 12.9 [9.9–15.9] | 1.0 [0–1.9] | 3.7 [2.1–5.3] |
| Women | 538 | 11.8 [8.7–15.0] | 39.2 [34.4–44.0] | 14.2 [10.7–17.7] | 21.3 [17.1–25.5] | 9.6 [6.7–12.5] | 0.6 [0–1.3] | 3.3 [1.8–4.7] |
|  |  |  |  |  |  |  |  |  |
| Occupational social grade |  |  |  |  |  |  |  |  |
| ABC1 (more advantaged) | 751 | 11.1 [8.7–13.6] | 40.2 [36.3–44.1] | 13.6 [10.8–16.3] | 19.5 [16.4–22.7] | 11.3 [8.7–13.9] | 0.9 [0.2–1.6] | 3.4 [1.9–4.9] |
| C2DE (less advantaged) | 516 | 12.3 [9.2–15.5] | 38.0 [33.2–42.8] | 12.9 [9.5–16.2] | 20.7 [16.7–24.7] | 11.7 [8.4–14.9] | 0.9 [0–1.8] | 3.5 [2.0–5.1] |
|  |  |  |  |  |  |  |  |  |
| Nation |  |  |  |  |  |  |  |  |
| England | 907 | 11.2 [9.1–13.6] | 39.2 [35.7–42.7] | 13.6 [11.3–16.3] | 19.9 [17.1–23.0] | 11.9 [9.7–14.5] | 1 [0.5–2] | 3.3 [2.2–4.7] |
| Wales | 117 | 18.8 [12.2–27.9] | 31.0 [22.5–41.0] | 11.2 [6.1–19.6] | 22.9 [15.7–32.3] | 10.6 [5.9–18.3] | 0 [0–0] | 5.4 [2.3–12.0] |
| Scotland | 243 | 14.6 [10.1–20.6] | 41.4 [34.8–48.3] | 9.8 [6.2–15.2] | 21.9 [16.7–28.2] | 7.6 [4.6–12.3] | 0 [0–0] | 4.8 [2.4–9.3] |
|  |  |  |  |  |  |  |  |  |
| History of ≥1 diagnosed mental health conditions^4^ |  |  |  |  |  |  |  |  |
| No | 412 | 10.9 [7.6–14.1] | 42.3 [37.0–47.6] | 10.8 [7.6–14.0] | 19.9 [15.5–24.2] | 10.4 [7.0–13.8] | 1.3 [0.1–2.5] | 4.4 [2.3–6.4] |
| Yes | 389 | 11.8 [8.5–15.0] | 40.8 [35.3–46.3] | 11.7 [8.1–15.3] | 21.2 [16.5–25.9] | 10.0 [6.7–13.3] | 1.1 [0–2.1] | 3.5 [1.6–5.5] |
|  |  |  |  |  |  |  |  |  |
| Smoking status |  |  |  |  |  |  |  |  |
| Long-term (≥1y) ex-smoker | 587 | 12.0 [9.0–15.0] | 45.0 [40.3–49.8] | 12.9 [9.6–16.1] | 21.3 [17.3–25.3] | 6.5 [4.1–9.0] | 0.5 [0–1.4] | 1.7 [0.3–3.1] |
| Recent (<1y) ex-smoker | 108 | 12.2 [5.8–18.5] | 35.5 [24.9–46.1] | 16.2 [7.4–25.0] | 20.8 [11.9–29.7] | 11.9 [5.2–18.5] | 1.6 [0–3.8] | 1.9 [0–4.7] |
| Current smoker | 459 | 10.1 [7.1–13.2] | 34.1 [29.0–39.2] | 12.5 [9.0–16.1] | 18.9 [14.8–22.9] | 16.0 [11.8–20.1] | 1.2 [0–2.4] | 7.1 [4.7–9.6] |
| Never smoker | 113 | 16.0 [7.8–24.1] | 32.5 [22.1–42.9] | 14.4 [7.2–21.5] | 19.4 [10.7–28.1] | 17.2 [9.5–24.9] | 0.6 [0–1.8] | 0 [0–0] |
|  |  |  |  |  |  |  |  |  |

^1^ Unweighted sample size.

^2^ Weighted row percentages.

^3^ Participants who described their gender in another way were excluded from analyses by gender due to low numbers.

^4^ Mental health conditions were not collected after June 2023, so results are based on aggregated data across January 2022 – June 2023.

##### Table S3. Usual nicotine strength used by adult (≥16y) vapers in Great Britain, January 2022-January 2024 – disposable device users

|  |  | **Nicotine strength, % [95% CI]^2^** | | | | | | |
| --- | --- | --- | --- | --- | --- | --- | --- | --- |
|  | ***N*^1^** | **No nicotine** | **6 mg/ml or less** | **7 to 11 mg/ml** | **12 to 19 mg/ml** | **20 mg/ml or more** | **Do not know if it contains nicotine** | **Contains nicotine but do not know the strength** |
|  |  |  |  |  |  |  |  |  |
| All adult vapers (≥16y) | 804 | 3.7 [2.4–5.0] | 23.3 [20.0–26.6] | 3.5 [2.1–5.0] | 5.3 [3.5–7.1] | 47.9 [44.0–51.8] | 1.2 [0.3–2.1] | 15.1 [12.4–17.8] |
|  |  |  |  |  |  |  |  |  |
| Vaping frequency |  |  |  |  |  |  |  |  |
| Non-daily | 184 | 4.2 [1.2–7.2] | 22.6 [15.7–29.5] | 3.6 [0.6–6.6] | 5.6 [2.1–9.2] | 40.1 [32.0–48.1] | 1.7 [0–3.6] | 22.2 [15.6–28.7] |
| Daily | 524 | 3.0 [1.5–4.5] | 23.8 [19.7–28.0] | 3.9 [1.9–5.8] | 5.6 [3.3–7.9] | 50.2 [45.3–55.1] | 0.8 [0–1.8] | 12.7 [9.5–15.8] |
|  |  |  |  |  |  |  |  |  |
| Age (years) |  |  |  |  |  |  |  |  |
| 16-24 | 341 | 1.2 [0.2–2.2] | 21.1 [16.0–26.1] | 3.1 [1.1–5.1] | 5.3 [2.4–8.1] | 58.1 [52.1–64.0] | 1.2 [0–2.5] | 10.1 [6.6–13.6] |
| 25-34 | 199 | 4.6 [1.6–7.6] | 25.7 [18.8–32.5] | 3.8 [0.7–6.9] | 4.5 [1.3–7.6] | 44.1 [36.1–52.0] | 1.4 [0–3.7] | 16.0 [10.4–21.6] |
| 35-44 | 112 | 6.7 [1.7–11.6] | 24.8 [15.3–34.3] | 5.3 [0.1–10.6] | 5.3 [0.6–10.0] | 43.0 [32.5–53.5] | 1.1 [0–2.8] | 13.8 [6.8–20.8] |
| 45-54 | 77 | 4.4 [0–8.8] | 24.6 [14.3–34.9] | 1.8 [0–4.5] | 7.5 [0.6–14.4] | 38.7 [25.7–51.8] | 0 [0–0] | 23.1 [11.6–34.6] |
| 55-64 | 50 | 11.4 [2.0–20.8] | 19.5 [7.9–31.1] | 3.3 [0–8.2] | 4.4 [0–10.5] | 25.9 [11.4–40.5] | 1.3 [0–4.0] | 34.1 [19.4–48.9] |
| ≥65 | 25 | 2.5 [0–7.6] | 35.3 [10.1–60.4] | 4.9 [0–14.6] | 10.3 [0–24.5] | 16.4 [0.1–32.7] | 3.3 [0–8.2] | 27.5 [8.4–46.5] |
|  |  |  |  |  |  |  |  |  |
| Gender^3^ |  |  |  |  |  |  |  |  |
| Men | 372 | 2.3 [0.9–3.8] | 21.8 [17.1–26.5] | 5.1 [2.6–7.7] | 6.1 [3.3–9.0] | 46.6 [40.7–52.4] | 1.1 [0–2.2] | 16.9 [12.6–21.2] |
| Women | 416 | 4.9 [2.8–7.0] | 24.9 [20.1–29.8] | 2.1 [0.6–3.7] | 4.3 [2.1–6.5] | 49.0 [43.6–54.5] | 1.3 [0–2.7] | 13.3 [9.9–16.8] |
|  |  |  |  |  |  |  |  |  |
| Occupational social grade |  |  |  |  |  |  |  |  |
| ABC1 (more advantaged) | 475 | 4.6 [2.6–6.6] | 21.9 [17.9–25.9] | 2.7 [1.1–4.3] | 5.2 [2.9–7.5] | 48.0 [43.2–52.9] | 0.6 [0–1.3] | 17.0 [13.3–20.6] |
| C2DE (less advantaged) | 329 | 3.0 [1.3–4.8] | 24.5 [19.3–29.6] | 4.2 [1.9–6.5] | 5.4 [2.8–8.0] | 47.8 [41.8–53.8] | 1.7 [0.2–3.2] | 13.5 [9.6–17.4] |
|  |  |  |  |  |  |  |  |  |
| Nation |  |  |  |  |  |  |  |  |
| England | 636 | 3.6 [2.5–5.3] | 23.8 [20.4–27.6] | 3.4 [2.2–5.4] | 5.5 [3.9–7.8] | 47.7 [43.5–52.0] | 1.1 [0.5–2.6] | 14.7 [12.1–17.9] |
| Wales | 51 | 4.0 [0.9–16.4] | 16.3 [8.4–29.3] | 2.5 [0.6–10.5] | 4.7 [1.0–19.6] | 59.5 [44.1–73.1] | 4.8 [1.3–16.1] | 8.0 [3.4–17.7] |
| Scotland | 117 | 4.8 [2.0–11.1] | 19.1 [12.3–28.4] | 4.8 [2.0–11.4] | 2.5 [0.9–7.0] | 45.5 [35.9–55.5] | 0.6 [0.1–3.9] | 22.6 [15.1–32.4] |
|  |  |  |  |  |  |  |  |  |
| History of ≥1 diagnosed mental health conditions^4^ |  |  |  |  |  |  |  |  |
| No | 219 | 5.1 [2.1–8.0] | 24.6 [18.2–31.1] | 5.1 [1.8–8.4] | 4.3 [1.4–7.3] | 44.0 [36.7–51.3] | 0.7 [0–1.8] | 16.1 [10.8–21.3] |
| Yes | 258 | 2.8 [0.9–4.7] | 24.2 [18.7–29.8] | 4.5 [1.7–7.3] | 6.0 [2.7–9.4] | 51.8 [45.1–58.4] | 2 [0.3–3.6] | 8.7 [5.0–12.3] |
|  |  |  |  |  |  |  |  |  |
| Smoking status |  |  |  |  |  |  |  |  |
| Long-term (≥1y) ex-smoker | 105 | 5.9 [1.3–10.4] | 19.7 [11.4–27.9] | 4.2 [0.3–8.2] | 7.5 [1.4–13.6] | 50.4 [39.5–61.3] | 0.3 [0–0.8] | 12.1 [5.1–19.0] |
| Recent (<1y) ex-smoker | 86 | 3.1 [0–6.3] | 26.9 [16.1–37.7] | 0 [0–0] | 2.1 [0–5.5] | 52.2 [39.7–64.7] | 2.7 [0–8.0] | 12.9 [4.8–21.1] |
| Current smoker | 464 | 3.5 [1.7–5.3] | 24.9 [20.3–29.5] | 4.2 [2.0–6.3] | 6.5 [4–9.1] | 44.0 [38.9–49.2] | 0.7 [0–1.4] | 16.2 [12.5–19.8] |
| Never smoker | 149 | 3.3 [0.6–5.9] | 18.8 [11.8–25.7] | 3.1 [0.4–5.8] | 1.9 [0–4.6] | 55.3 [46.4–64.1] | 2.5 [0–5.1] | 15.2 [8.8–21.5] |
|  |  |  |  |  |  |  |  |  |

^1^ Unweighted sample size.

^2^ Weighted row percentages.

^3^ Participants who described their gender in another way were excluded from analyses by gender due to low numbers.

^4^ Mental health conditions were not collected after June 2023, so results are based on aggregated data across January 2022 – June 2023.

##### Table S4. Usual nicotine strength used by adult (≥16y) vapers in Great Britain, January 2022-January 2024 – pod device users

|  |  | **Nicotine strength, % [95% CI]^2^** | | | | | | |
| --- | --- | --- | --- | --- | --- | --- | --- | --- |
|  | ***N*^1^** | **No nicotine** | **6 mg/ml or less** | **7 to 11 mg/ml** | **12 to 19 mg/ml** | **20 mg/ml or more** | **Do not know if it contains nicotine** | **Contains nicotine but do not know the strength** |
|  |  |  |  |  |  |  |  |  |
| All adult vapers (≥16y) | 262 | 9.2 [6.1–13.7] | 18.4 [13.7–24.2] | 9.8 [6.3–14.8] | 27.1 [21.7–33.4] | 16.3 [11.8–22.0] | 2.0 [0.9–4.5] | 17.2 [12.3–23.4] |
|  |  |  |  |  |  |  |  |  |
| Vaping frequency |  |  |  |  |  |  |  |  |
| Non-daily | 57 | 15.9 [4.8–26.9] | 17.2 [6.7–27.8] | 8.6 [0–18.3] | 21.9 [10.6–33.2] | 5.3 [0–11.7] | 2.0 [0–5.1] | 29.0 [14.2–43.9] |
| Daily | 175 | 7.2 [3.4–11.1] | 17.2 [11.1–23.4] | 10.3 [5.4–15.3] | 31.6 [23.9–39.3] | 19.8 [13.0–26.5] | 1.4 [0–3.2] | 12.4 [7.3–17.5] |
|  |  |  |  |  |  |  |  |  |
| Age (years) |  |  |  |  |  |  |  |  |
| 16-24 | 46 | 7.6 [2.7–19.3] | 14.5 [6.6–29.0] | 10.0 [4.0–23.1] | 15.2 [6.8–30.5] | 33.1 [19.9–49.5] | 4.2 [1.0–16.2] | 15.4 [6.9–30.7] |
| 25-34 | 51 | 2.8 [0.7–10.9] | 15.3 [6.8–30.9] | 11.1 [4.6–24.5] | 24.3 [13.8–39.2] | 19.6 [10.1–34.6] | 1.6 [0.2–11.1] | 25.3 [13.6–42.1] |
| 35-44 | 51 | 10.4 [4.2–23.5] | 23.7 [13.4–38.4] | 18.1 [8.3–35.1] | 23.2 [13.0–37.8] | 8.8 [3.0.–22.7] | 0 [0–0] | 15.9 [6.9–32.5] |
| 45-54 | 37 | 14.6 [5.9–31.9] | 23.1 [11.0–42.3] | 7.9 [2.6–21.2] | 34.4 [20.1–52.2] | 7.4 [2.1–22.7] | 1.0 [0.1–7.7] | 11.6 [3.5–32.1] |
| 55-64 | 39 | 21.8 [9.7–42.0] | 14.7 [5.8–32.6] | 2.3 [0.3–15.8] | 38.7 [23.2–56.9] | 14.2 [5.7–31.1] | 0 [0–0] | 8.3 [3.1–20.4] |
| ≥65 | 38 | 4.4 [1.0–17.9] | 20.6 [9.3–39.7] | 1.3 [0.2–9.3] | 39.5 [23.0–58.7] | 7.5 [2.3–21.9] | 6.6 [1.9–20.3] | 20.1 [9.2–38.5] |
|  |  |  |  |  |  |  |  |  |
| Gender^3^ |  |  |  |  |  |  |  |  |
| Men | 121 | 5.9 [2.9–11.6] | 22.5 [15.1–32.2] | 8.3 [4.1–16.0] | 26.6 [19.0–35.9] | 17.1 [10.6–26.5] | 2.6 [0.9–7.4] | 16.9 [10.0–27.2] |
| Women | 134 | 10.8 [6.1–18.3] | 14.7 [9.4–22.3] | 11.6 [6.6–19.6] | 28.3 [20.6–37.5] | 16.2 [10.5–24.3] | 0.9 [0.2–4.0] | 17.5 [11.2–26.3] |
|  |  |  |  |  |  |  |  |  |
| Occupational social grade |  |  |  |  |  |  |  |  |
| ABC1 (more advantaged) | 163 | 9.7 [5.8–15.9] | 19.9 [13.8–27.7] | 8.3 [4.9–13.8] | 26.3 [19.8–34.1] | 18.7 [12.7–26.6] | 1.8 [0.5–5.9] | 15.4 [10.4–22.1] |
| C2DE (less advantaged) | 99 | 8.8 [4.5–16.3] | 16.8 [10.2–26.3] | 11.3 [5.8–20.8] | 28.0 [19.5–38.4] | 13.8 [7.9–23.0] | 2.3 [0.8–6.5] | 19.0 [11.2–30.4] |
|  |  |  |  |  |  |  |  |  |
| Nation |  |  |  |  |  |  |  |  |
| England | 214 | 9.6 [6.3–14.4] | 17.9 [13–24.2] | 9.2 [5.6–14.6] | 26.3 [20.5–33.0] | 17.5 [12.6–23.6] | 1.9 [0.8–4.6] | 17.7 [12.5–24.4] |
| Wales | 16 | 14.3 [2.6–50.6] | 34.5 [10.9–69.4] | 25.6 [7.7–58.7] | 15.0 [3.8–44.1] | 3.7 [0.4–26.2] | 0 [0–0] | 6.9 [1.4–28.5] |
| Scotland | 32 | 0 [0–0] | 16.5 [6.4–36.6] | 11.4 [3.9–28.9] | 50.0 [31.3–68.7] | 2.9 [0.4–19.0] | 5.6 [1.3–21.0] | 13.7 [5.3–30.9] |
|  |  |  |  |  |  |  |  |  |
| History of ≥1 diagnosed mental health conditions^4^ |  |  |  |  |  |  |  |  |
| No | 86 | 13.4 [7.6–22.5] | 19.3 [11.7–30.2] | 7.3 [2.9–17.1] | 20.9 [13.3–31.3] | 16.5 [9.6–26.8] | 2.8 [0.9–8.7] | 19.7 [11.2–32.4] |
| Yes | 86 | 4.6 [1.7–12.1] | 19.8 [12.3–30.3] | 12.1 [6.0–23.2] | 35.7 [25.7–47.2] | 14.2 [8.0–24.0] | 0.9 [0.1–6.5] | 12.6 [7.2–21.1] |
|  |  |  |  |  |  |  |  |  |
| Smoking status |  |  |  |  |  |  |  |  |
| Long-term (≥1y) ex-smoker | 80 | 12.8 [6.8–22.7] | 14.1 [7.8–24.2] | 9.6 [4.1–20.8] | 40.3 [28.9–53.0] | 10.9 [5.3–21.0] | 0.4 [0.1–3.2] | 11.8 [5.4–23.9] |
| Recent (<1y) ex-smoker | 25 | 7.5 [1.7–27.5] | 34.9 [16.4–59.3] | 14.2 [4.8–35.2] | 28.6 [13.3–51.2] | 5.2 [0.7–31.4] | 4.8 [0.6–29.3] | 4.7 [0.6–29.1] |
| Current smoker | 132 | 7.3 [3.6–14.1] | 18.6 [12.2–27.4] | 10.2 [5.4–18.3] | 20.5 [14.2–28.8] | 19.7 [13.1–28.6] | 1.5 [0.5–5] | 22.2 [14.8–31.9] |
| Never smoker | 25 | 10.9 [3.3–30.7] | 13.9 [4.9–33.7] | 3.9 [0.8–16.7] | 22.1 [8.9–45.4] | 24.4 [9.9–48.6] | 6.8 [1.5–25.7] | 18.0 [6.0–42.9] |
|  |  |  |  |  |  |  |  |  |

^1^ Unweighted sample size.

^2^ Weighted row percentages.

^3^ Participants who described their gender in another way were excluded from analyses by gender due to low numbers.

^4^ Mental health conditions were not collected after June 2023, so results are based on aggregated data across January 2022 – June 2023.
